# Exploring the Interplay of Muscular Endurance, Functional Balance, and Limits of Stability: A Comparative Study in Individuals with Lumbar Spondylosis Using a Computerized Stabilometric Force Platform

**DOI:** 10.3390/life13102104

**Published:** 2023-10-23

**Authors:** Fareed F. Alfaya, Ravi Shankar Reddy, Mastour Saeed Alshahrani, Ajay Prashad Gautam, Debjani Mukherjee, Zuhair A. Al Salim, Raee S. Alqhtani, Hussain Saleh H. Ghulam, Abdullah Mohammed Alyami, Saeed Al Adal, Abdullah Ali Jabour

**Affiliations:** 1 Department of Orthopedic Surgery, College of Medicine, King Khalid University, Abha 61421, Saudi Arabia; ffalfaia@kku.edu.sa; 2 Department of Medical Rehabilitation Sciences, College of Applied Medical Sciences, King Khalid University, Abha 61421, Saudi Arabia; msdalshahrani@kku.edu.sa (M.S.A.); agautam@kku.edu.sa (A.P.G.); debjani@kku.edu.sa (D.M.); 3 Department of Sport Science and Physical Activity, College of Science, University of Hafr Al Batin, Hafr Al Batin 39524, Saudi Arabia; drzuhair@uhb.edu.sa; 4 Physical Therapy Department, Medical Applied Sciences College, Najran University, Najran 66462, Saudi Arabia; rsalhyani@nu.edu.sa (R.S.A.); hsghulam@nu.edu.sa (H.S.H.G.); amalkhuriem@nu.edu.sa (A.M.A.); syaladel@nu.edu.sa (S.A.A.); 5 Abu-Arish General Hospital, Ministry of Health, Jizan 84421, Saudi Arabia; ajabour@moh.gov.sa

**Keywords:** lumbar spondylosis, lumbar extensor endurance, functional balance, limits of stability, musculoskeletal function, rehabilitation

## Abstract

Lumbar spondylosis, characterized by degenerative changes in the lumbar spine, often leads to pain, reduced spinal stability, and musculoskeletal dysfunction. Understanding the impact of lumbar spondylosis on musculoskeletal function, particularly lumbar extensor endurance, functional balance, and limits of stability, is crucial for improving the management and well-being of affected individuals. This study aimed to assess lumbar extensor endurance, functional balance, and limits of stability in individuals with lumbar spondylosis compared to age-matched healthy individuals and explore the correlations among these parameters within the lumbar spondylosis group. The lumbar spondylosis group consisted of 60 individuals initially screened by an orthopedician and referred to physical therapy. Age-matched healthy controls (*n* = 60) were recruited. Inclusion criteria encompassed adults aged 45–70 years for both groups. Lumbar extensor endurance was assessed using the Sorensen test, functional balance with the Berg Balance Scale, and limits of stability using a computerized stabilometric force platform. Lumbar extensor endurance was significantly lower in individuals with lumbar spondylosis compared to healthy controls (23.06 s vs. 52.45 s, *p* < 0.001). Functional balance, as assessed by the Berg Balance Scale, demonstrated a significant decrement in the lumbar spondylosis group (48.36 vs. 53.34, *p* < 0.001). Additionally, limits of stability variables, under both eyes-open and eyes-closed conditions, exhibited marked impairments in the lumbar spondylosis group (*p* < 0.001 for all variables). Within the lumbar spondylosis group, lumbar extensor endurance exhibited significant positive correlations with functional balance (0.46, *p* < 0.001) and negative correlations with limits of stability variables (r ranging from −0.38 to −0.49, *p* < 0.01 for all variables). This study underscores the significance of addressing lumbar extensor endurance, functional balance, and stability impairments in the comprehensive management of lumbar spondylosis.

## 1. Introduction

Lumbar spondylosis is a prevalent degenerative condition of the lumbar spine, characterized by progressive structural changes in the intervertebral discs, facet joints, and vertebral bodies [1]. It is a common cause of low back pain and disability, affecting millions of individuals worldwide [2]. The pathophysiology of lumbar spondylosis involves multifactorial processes, including age-related degeneration, genetic predisposition, and mechanical stresses on the lumbar spine [3,4,5,6]. These processes often result in the loss of spinal stability, which can significantly impact an individual’s functional balance and overall quality of life [7].

Lumbar extensor endurance plays a pivotal role in maintaining spinal stability and overall musculoskeletal function [8,9]. These muscles, located in the lower back, are responsible for keeping the spine upright, stabilizing it during various activities, and facilitating trunk and pelvic movements [8,9]. Adequate lumbar extensor endurance is essential for maintaining good posture, preventing excessive stress on the spine, and ensuring optimal spinal alignment [8,9]. Impairments in lumbar extensor endurance have been strongly associated with low back pain, a prevalent and debilitating condition that affects millions of individuals worldwide [8,9]. Weakness or dysfunction in these muscles can lead to poor posture, reduced spinal stability, and an increased risk of low back pain [8,9,10]. Lumbar extensor muscles play a vital role in maintaining an upright posture, stabilizing the spine, and facilitating movements of the trunk and pelvis [11,12]. Dysfunction of the lumbar extensor muscles can lead to impaired balance and reduced limits of stability, which may increase the risk of falls and further exacerbate the disability associated with lumbar spondylosis [13,14]. The assessment of lumbar extensor endurance, functional balance, and limits of stability is of paramount importance in understanding the functional limitations and rehabilitation needs of individuals with lumbar spondylosis with low back pain [15,16].

In the context of lumbar spondylosis, the assessments used to evaluate various aspects of physical function play a pivotal role in understanding the condition’s impact on individuals. One such assessment, the Sorensen test, also known as the Biering-Sorensen test, stands out as a reliable and widely recognized measurement [17]. It enables the quantification of the endurance capacity of the lumbar extensor muscles, making it a valuable tool for assessing spinal stability and low back health in individuals affected by lumbar spondylosis [18]. Additionally, the evaluation of functional balance is a crucial aspect of comprehending the challenges faced by these individuals in their daily lives [19]. The Berg Balance Scale (BBS), a well-established assessment, allows for the systematic evaluation of an individual’s ability to maintain stability during various routine activities, providing essential insights into their overall balance and the risk of falls [20]. Furthermore, the assessment of limits of stability employs a computerized stabilometric force platform, which aids in quantifying an individual’s capacity to shift their center of pressure (COP) within their base of support [21,22]. This comprehensive assessment provides a detailed understanding of postural control and stability in both static and dynamic conditions, further enhancing our ability to address the functional limitations associated with lumbar spondylosis.

To achieve these objectives, we first seek to assess and compare lumbar extensor endurance, functional balance, and limits of stability between individuals with lumbar spondylosis and their age-matched healthy counterparts. This comparison is crucial to establish the extent of functional deficits in the lumbar spondylosis population. Understanding the differences between these groups will help identify specific impairments associated with lumbar spondylosis, providing valuable insights into the functional consequences of the condition [23]. Moreover, by exploring the correlations between lumbar extensor endurance, functional balance, and limits of stability in individuals with lumbar spondylosis, we aim to unravel the intricate relationships among these variables [24]. Such correlations can shed light on the underlying mechanisms that connect muscular endurance, postural control, and stability in this population [24]. For instance, we hypothesize that individuals with lower lumbar extensor endurance may exhibit poorer functional balance and reduced limits of stability, highlighting the significance of these muscles in maintaining postural control and stability.

The rationale for this study is rooted in the need to address the functional limitations experienced by individuals with lumbar spondylosis comprehensively. While the condition’s structural changes in the lumbar spine are well-documented, the impact of these changes on functional outcomes such as balance and stability remains less explored [25]. As key players in spinal stability, the lumbar extensor muscles present a promising avenue for investigation [26]. Recognizing their role in maintaining postural control and balance is essential for the development of effective rehabilitation strategies tailored to the unique needs of individuals with lumbar spondylosis [27]. Understanding the relationship between lumbar extensor endurance, functional balance, and limits of stability in individuals with lumbar spondylosis is of great clinical significance [28]. Previous research has shown that lumbar extensor muscle weakness and dysfunction are common findings in individuals with lumbar spondylosis [15,29]. However, the extent to which these muscular impairments contribute to functional deficits in balance and stability remains to be fully elucidated.

This study seeks to bridge the gap in knowledge by assessing and comparing lumbar extensor endurance, functional balance, and limits of stability between individuals with lumbar spondylosis and age-matched healthy individuals. Additionally, we aim to investigate the potential correlations between these variables in individuals with lumbar spondylosis. By doing so, we hope to provide valuable insights into the biomechanical and neuromuscular factors that influence functional outcomes in this population. In summary, this research endeavors to comprehensively assess the relationship between lumbar extensor endurance, functional balance, and limits of stability in individuals with lumbar spondylosis. Achieving these objectives will contribute to a deeper understanding of the functional limitations associated with lumbar spondylosis and may inform the development of targeted rehabilitation strategies to improve the quality of life for affected individuals.

## 2. Materials and Methods

### 2.1. Design, Settings, and Ethics

This comparative cross-sectional study was conducted between January 2020 and March 2023 in the medical rehabilitation clinics of King Khalid University. The study design aimed to assess and compare lumbar extensor endurance, functional balance, and limits of stability between individuals with lumbar spondylosis and age-matched healthy individuals. The ethical approval for this study was obtained from the [KKU, Research Ethics Committee]. The approval code is [REC # 14/22/456]. The research adhered to ethical principles outlined in the Declaration of Helsinki and received approval from the institutional ethics committee. Informed consent was obtained from all participants prior to their inclusion in the study, ensuring their voluntary participation and confidentiality of data.

### 2.2. Participants

In this study, participants were recruited based on specific inclusion and exclusion criteria to ensure the relevance of the findings. In the lumbar spondylosis group, participants met the inclusion criteria if they had received a lumbar spondylosis diagnosis from an orthopedician, which was further substantiated by radiological imaging. Radiological criteria for lumbar spondylosis encompass joint space narrowing, osteophyte formation, subchondral sclerosis, and the presence of cysts [30,31]. The age range for inclusion in this group was set between 40 and 70 years to match the age-matched healthy individuals who served as the control group. All participants were required to provide informed consent and express a willingness to participate in the study. Additionally, participants in the lumbar spondylosis group were excluded if they had other significant spinal pathologies, severe neurological deficits, concurrent medical conditions, or musculoskeletal disorders that could potentially confound the study results. Furthermore, individuals with a history of recent spine surgery or major musculoskeletal injuries that could affect the study outcomes were also excluded. It was important that all participants in this group had the ability to tolerate physical therapy sessions and participate in the prescribed assessments.

For the age-matched healthy group, inclusion criteria were defined as individuals aged between 40 and 70 years with no history of lumbar spine disorders or chronic low back pain. Exclusion criteria for this group encompassed any significant musculoskeletal or neurological disorders that could potentially impact their balance, stability, or endurance. Additionally, individuals in this group were excluded if they had diabetes mellitus, cognitive impairment, or vestibular disorders that might impair physical performance or confound the study outcomes. Like the lumbar spondylosis group, participants in the healthy control group needed to provide informed consent and demonstrate their willingness to participate. All participants in this group were also required to have the ability to understand and follow study instructions, as well as tolerate the physical assessments and exercises.

To ensure a rigorous screening process, individuals diagnosed with lumbar spondylosis underwent initial evaluation by an orthopedician who confirmed the diagnosis through clinical assessment and radiological findings. The exclusion criteria for this group were twofold: first, any substantial musculoskeletal or neurological disorders that could potentially influence their balance, stability, or endurance were grounds for exclusion. Second, individuals were excluded from this group if they had diabetes mellitus, cognitive impairment, or vestibular disorders that might hinder their physical performance or introduce confounding factors into the study results. On the other hand, age-matched healthy individuals were recruited from the community through advertisements and underwent a thorough screening process to confirm their eligibility based on the defined criteria. The recruitment process for both groups prioritized the principles of informed consent, privacy, and data confidentiality, aligning with ethical guidelines and the principles outlined in the Declaration of Helsinki.

### 2.3. Outcome Measures

In this study, a comprehensive battery of outcome measures was employed to assess lumbar extensor endurance, functional balance, and limits of stability in both the lumbar spondylosis and age-matched healthy groups. These outcome measures were selected to provide a multidimensional evaluation of the participants’ physical function and to address the study objectives.

#### 2.3.1. Lumbar Extensor Endurance

**Figure 1:**
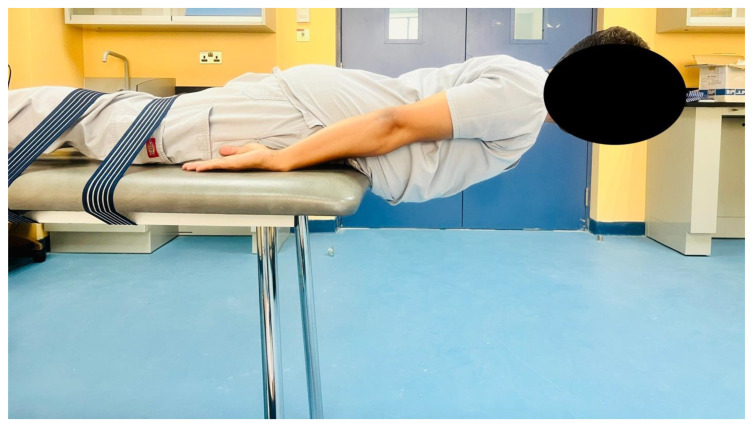
Lumbar extensor endurance measurement using the Sorensen test (trunk extensor endurance test).

Lumbar extensor endurance, a pivotal aspect of this study’s assessment protocol, was evaluated using the Sorensen test, also known as the trunk extensor endurance test [32]. This assessment is well-established and exhibits a high level of reliability (ICC [1,1] = 0.88; 95% CI, 0.73–0.95) when measuring the endurance capacity specific to the lumbar extensor muscles [33]. Participants were positioned in a prone (face-down) orientation on an examination table, ensuring that their upper body remained unsupported (Figure 1).

This positioning was achieved by aligning the upper edge of the table just below the participants’ hip joints, thus allowing the upper body to hang freely over the table’s edge. Their feet were securely anchored or held down to eliminate unintended movement during the test, ensuring that the lumbar extensors were the primary muscles engaged. The test commenced with standardized instructions provided to all participants. They were directed to lift their upper body, encompassing their chest and shoulders, while maintaining a horizontal position of their trunk. This required participants to form a straight line extending from their head to their heels, effectively assuming a “plank” position. The elevation of the upper body was achieved through the activation of the lumbar extensor muscles. Participants were instructed to sustain this contraction for as long as possible. To objectively quantify lumbar extensor endurance, a digital timer was employed to record the duration in seconds that participants could maintain the lifted position. Timing commenced as soon as participants initiated the elevation of their upper body and ceased when they were no longer able to maintain the horizontal trunk position, primarily due to muscle fatigue or discomfort.

The Sorensen test offered a quantitative measurement of lumbar extensor endurance, permitting a direct comparison of this parameter between individuals in both the lumbar spondylosis and age-matched healthy groups [32,34,35]. Standardized instructions, blinded assessments by trained physical therapists, and documentation of discontinuations due to discomfort or fatigue were implemented to ensure consistency, minimize potential bias, and maintain the reliability of the test.

#### 2.3.2. Functional Balance

The assessment of functional balance was conducted using the Berg Balance Scale (BBS), a widely recognized and validated clinical tool that offers a comprehensive evaluation of an individual’s balance during various functional tasks [36,37]. The selection of the BBS was made based on its established reliability and practical relevance to daily activities. This assessment consisted of 14 specific tasks designed to simulate real-world balance challenges. Participants were instructed to perform these tasks according to standardized guidelines provided by trained physical therapists [37]. Each task was scored based on the participant’s performance, utilizing a rating scale that ranged from 0 to 4 points [37]. A score of 0 indicated the inability to perform the task, while a score of 4 signified independent and safe task completion. Scores of 1, 2, and 3 represented varying degrees of assistance or difficulty encountered during task execution. The individual task scores were then summed to obtain a total BBS score for each participant, with the maximum achievable score being 56. The tasks included in the BBS assessment encompassed a diverse range of functional movements, such as sitting to standing, reaching, turning, and stepping. These tasks aimed to replicate the balance challenges encountered in daily life, providing a holistic assessment of an individual’s functional balance capabilities. Importantly, the BBS allowed for the quantification of participants’ balance performance, enabling the comparison of functional balance between the lumbar spondylosis group and the age-matched healthy group.

To maintain consistency and objectivity in the assessment process, trained physical therapists administered the BBS assessments while being blinded to the participants’ group assignments. Any potential variations in the interpretation of task performance were minimized through standardized instructions and scoring criteria. The choice of the BBS as the assessment tool for functional balance was driven by its clinical relevance and its ability to provide valuable insights into the impact of lumbar spondylosis on an individual’s ability to perform daily activities safely and independently.

#### 2.3.3. Limits of Stability Assessment

The assessment of limits of stability (LOS) was conducted using the Iso-Free Balance System, a highly regarded computerized stabilometric force platform [38]. The system offers precise and objective quantification of postural control and stability across various planes [28]. Stringent standardization procedures were meticulously followed to ensure the reliability and reproducibility of this assessment [39,40]. Notably, the limits of stability test demonstrated its reliability and validity, as reflected in intraclass correlation coefficient (ICC) values ranging from 0.82 to 0.48, indicating strong to moderate reliability [39,40].

**Figure 2:**
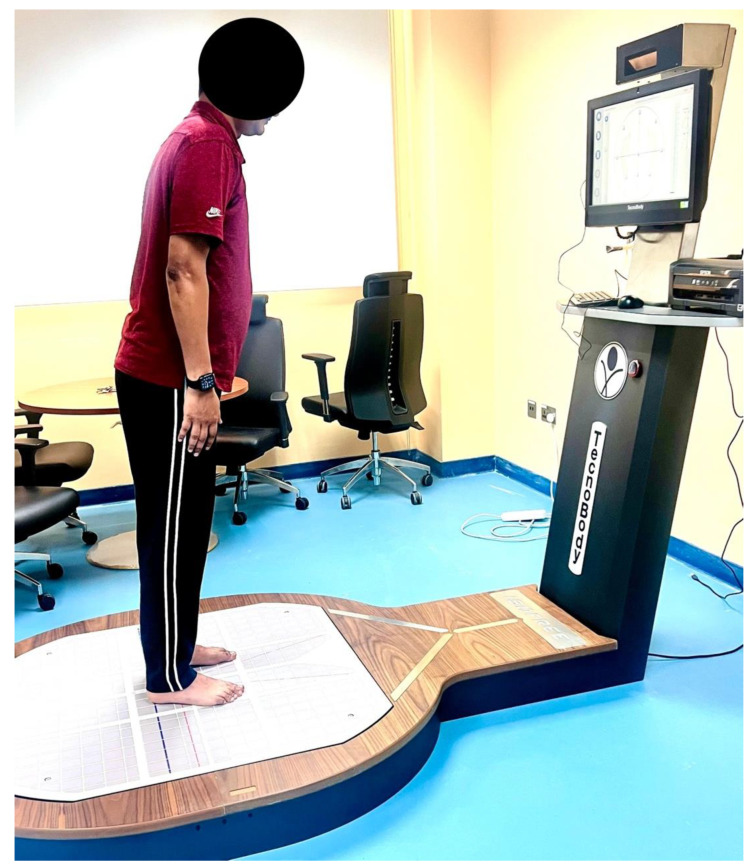
Limits of stability assessment using computerized stabilometric force platform.

Participants were instructed to stand in a relaxed, barefoot position on the force platform with their heels aligned to a designated reference line, and their arms held comfortably at their sides. During the test, they fixated their gaze on the target mark “X” displayed on a computer monitor (Figure 2) in the eyes-open condition and closed their eyes in the eyes-closed condition. Participants were then challenged to maintain their balance for a duration of thirty seconds. Posturography measurements encompassed essential balance metrics, including anterior to posterior (A–P) sway (in millimeters, mm), medial to lateral (M–L) sway (in mm), and the ellipse area of the COP in square millimeters (mm^2^) within this timeframe. A 30 s resting interval allowed participants to stand comfortably between trials, which were repeated three times, with subsequent analysis based on the most successful attempt.

The variables related to limits of stability, which offer valuable insights into postural stability, were determined from COP displacements recorded during the limits of stability assessment. These variables include:

1. Ellipse Area (mm^2^): This metric quantifies the area enclosed by the trajectory of the COP during the limits of stability test. It reflects how effectively an individual can shift their body weight within their base of support. Larger Ellipse Area values indicate greater COP displacement and signify challenges in maintaining postural stability.
2. A–P Sway (mm): A–P sway measures anterior–posterior COP and body sway. It assesses an individual’s control over forward and backward movements. Increased A–P sway values indicate heightened instability along the sagittal plane.
3. M–L Sway (mm): M–L sway quantifies medial–lateral COP and body sway, evaluating control over lateral movements. Elevated M–L sway values denote increased instability along the frontal plane.

### 2.4. Sample Size Calculation

The determination of our study’s sample size drew inspiration from Behennah et al.’s [41] research on extensor muscle strength, where a moderate effect size of approximately 0.60 was reported. Considering the analogous nature of our study, focusing on lumbar extensor endurance and related parameters, we used this estimated effect size as a foundation for our sample size calculation. Our goal was to attain a statistical power of 0.80, indicating an 80% likelihood of detecting true differences while maintaining a significance level of 0.05. Based on these parameters and the estimated effect size, our sample size calculation confirmed the need for approximately 60 participants in each group. This sample size not only resonated with the findings of Behennah et al. [41] but also fortified our study’s ability to robustly discern significant disparities between the lumbar spondylosis and age-matched healthy control groups regarding lumbar extensor endurance and related parameters.

### 2.5. Data Analysis

The data analysis for this study was conducted with the assumption of normal distribution, which was confirmed by performing Shapiro–Wilk tests for normality on the collected data. These tests provided statistical evidence supporting the normal distribution of the data. Descriptive statistics, including means and standard deviations, were computed for lumbar extensor endurance, functional balance (assessed by the BBS), and limits of stability (measured using the Biodex Balance System) in both the lumbar spondylosis and age-matched healthy control groups. To address the primary research objectives, independent *t*-tests were employed to compare these measures between the two groups. Additionally, Pearson correlation coefficients were calculated to explore potential associations within the lumbar spondylosis group, specifically between lumbar extensor endurance and functional balance, as well as between lumbar extensor endurance and limits of stability. A significance level (alpha) of 0.05 was maintained for all analyses, ensuring statistical rigor. The choice of statistical methods aligned with data distribution characteristics and research objectives, facilitating a comprehensive examination of lumbar extensor function, balance, and stability in individuals with lumbar spondylosis.

## 3. Results

Table 1 presents the physical and demographic characteristics of the study participants, distinguishing between the lumbar spondylosis group (n = 60) and the healthy control group (n = 60). The analysis revealed that there were no statistically significant differences between the two groups in terms of age (*p* = 0.826), sex distribution (*p* = 0.975), or BMI (*p* = 0.416). However, it is important to note that the lumbar spondylosis group reported a mean duration of symptoms of 12.33 months, accompanied by a pain intensity of 57.66 on the VAS scale and a functional disability score of 22.63 on the Oswestry Disability Index (ODI) scale, while these parameters were not applicable to the healthy control group.

Table 2 presents the comparisons of lumbar extensor endurance, functional balance, and limits of stability variables between the lumbar spondylosis group (n = 60) and the healthy control group (n = 60).

In terms of lumbar extensor endurance, the lumbar spondylosis group exhibited a significantly lower mean endurance time (23.06 s ± 8.38) compared to the healthy control group (52.45 s ± 11.48), with a *p*-value of <0.001, signifying a substantial difference. For functional balance, as assessed by BBS scores (ranging from 0 to 56), the lumbar spondylosis group had a mean score of 48.36 (±3.26), while the healthy control group scored higher at 53.34 (±2.48). This difference was statistically significant with a *p*-value of <0.001.

Regarding the limits of stability variables, the analysis was performed under two conditions: eyes open and eyes closed. Under eyes-open conditions, the lumbar spondylosis group exhibited a larger ellipse area (337.07 mm^2^ ± 128.12), increased anterior–posterior sway (6.43 mm^2^ ± 2.16), and medial–lateral sway (3.38 mm^2^ ± 0.89) compared to the healthy control group, which recorded values of 176.94 mm^2^ ± 63.87, 4.23 mm^2^ ± 1.01, and 2.12 mm^2^ ± 0.32, respectively. These differences were highly significant (*p* < 0.001) for all variables.

Under eyes-closed conditions, similar trends were observed. The lumbar spondylosis group exhibited a larger ellipse area (512.38 mm^2^ ± 110.26), increased anterior–posterior sway (8.32 mm ± 2.92), and medial–lateral sway (5.23 mm ± 1.96) compared to the healthy control group, which recorded values of 256.12 mm^2^ ± 98.87, 5.12 mm^2^ ± 2.08, and 3.99 mm^2^ ± 1.98, respectively. Again, these differences were highly significant (*p* < 0.001) for all variables.

Table 3 presents the results of correlation analyses conducted within the lumbar spondylosis group, examining the relationships between lumbar extensor endurance and various parameters related to functional balance and limits of stability.

Our results revealed significant positive correlations between lumbar extensor endurance and functional balance (r = 0.46, *p* < 0.01), indicating that individuals with greater lumbar extensor endurance tended to have better functional balance as assessed by the BBS. Additionally, we found negative correlations between lumbar extensor endurance and limits of stability variables under both eyes-open and eyes-closed conditions, signifying those individuals with higher lumbar extensor endurance exhibited improved postural control and stability. Specifically, under eyes-open conditions, we observed negative correlations between lumbar extensor endurance and ellipse area (r = −0.38, *p* < 0.01), A–P sway (r = −0.41, *p* < 0.01), and M–L sway (r = −0.43, *p* < 0.01). Under eyes-closed conditions, lumbar extensor endurance exhibited negative correlations with ellipse area (r = −0.49, *p* < 0.01), A–P sway (r = −0.47, *p* < 0.01), and M–L sway (r = −0.45, *p* < 0.01). These findings emphasize the critical role of lumbar extensor muscles in maintaining postural control and balance in lumbar spondylosis patients. The correlations identified in this study provide valuable insights that can inform the development of targeted rehabilitation strategies to enhance the overall well-being of individuals affected by lumbar spondylosis.

## 4. Discussion

This study aimed to comprehensively assess lumbar extensor endurance, functional balance, and limits of stability in individuals diagnosed with lumbar spondylosis, comparing them to age-matched healthy individuals. The findings revealed marked differences between the two groups. Individuals with lumbar spondylosis exhibited significantly lower lumbar extensor endurance and compromised functional balance, as indicated by the BBS scores. Additionally, their limits of stability, assessed under various conditions, showed substantial impairments when compared to healthy controls. Within the lumbar spondylosis group, positive correlations were observed between lumbar extensor endurance and parameters related to functional balance, and negative correlations with limits of stability. These results highlight the comprehensive impact of lumbar spondylosis on musculoskeletal function and suggest potential avenues for targeted rehabilitation strategies to enhance both balance and stability in affected individuals.

The substantial reduction in lumbar extensor endurance observed in the lumbar spondylosis group is supported by a body of prior research, consistently reporting compromised lumbar extensor function in individuals with various lumbar spine pathologies, including spondylosis [42,43]. Lumbar spondylosis is characterized by degenerative changes in the spinal structures, including the intervertebral discs and facet joints. These degenerative changes can result in pain, reduced spinal stability, and altered biomechanics, all of which contribute to decreased lumbar extensor endurance. This reduction in endurance is not only a consequence of the condition but also a factor that exacerbates functional limitations in individuals with lumbar spondylosis. Numerous studies have substantiated the association between lumbar spine pathologies and diminished lumbar extensor endurance [44,45,46,47,48]. For instance, a study by Ito et al. [15] found that patients with chronic low back pain due to lumbar spondylosis exhibited reduced lumbar extensor muscle endurance compared to healthy individuals. This reduction in endurance was attributed to muscle deconditioning, pain-related inhibition of muscle function, and muscle imbalances around the lumbar spine [49]. Furthermore, Matheve et al. [50] highlighted the inhibitory effects of pain on the deep lumbar extensors, leading to impaired endurance. This inhibition may be particularly pronounced in individuals with lumbar spondylosis due to the chronic nature of the condition [51].

Additionally, muscle weakness in the lumbar extensors, which is often observed in individuals with lumbar spondylosis, contributes to decreased endurance [52]. Jahandideh et al. [53] demonstrated that lumbar extensor muscle weakness was prevalent in patients with chronic low back pain, a finding that aligns with the muscle imbalances commonly seen in lumbar spondylosis patients. These findings underscore the clinical relevance of lumbar extensor endurance as a key parameter affected by lumbar spondylosis. Addressing this impairment through targeted interventions may hold the potential to improve functional outcomes and enhance the quality of life for individuals with lumbar spondylosis. However, it is crucial to acknowledge the limitations of this study, consider the multifaceted nature of lumbar spondylosis, and continue exploring effective rehabilitation strategies in future research.

The significant decrement in functional balance observed in individuals with lumbar spondylosis is supported by several justifications and aligns with findings from previous studies in the field [15,54]. Functional balance is a critical aspect of daily life, encompassing the ability to maintain stability during various activities, including standing, walking, and transitioning between positions [55]. Lumbar spondylosis, characterized by degenerative changes in the lumbar spine, including intervertebral disc degeneration and facet joint alterations, has a profound impact on musculoskeletal function [56]. These degenerative changes often lead to chronic pain, reduced spinal stability, and biomechanical alterations, all of which significantly compromise functional balance [56]. The association between lumbar spine disorders and impaired functional balance is well-documented [56,57]. Individuals with lumbar spondylosis frequently experience difficulties in maintaining equilibrium due to pain and decreased lumbar extensor muscle strength [54]. Previous research, including the study by Ito et al. [15], has consistently demonstrated weakened lumbar extensor muscles in patients with chronic low back pain, a condition closely related to lumbar spondylosis [58]. This muscular weakness can impede the ability to stabilize the lumbar spine during dynamic activities, such as maintaining balance [58,59]. Moreover, pain-related inhibitions play a pivotal role in functional balance impairments [60,61]. Pain associated with lumbar spondylosis can lead to altered movement patterns, muscle recruitment, and compensatory strategies aimed at minimizing discomfort [62]. The study by Porwal et al. [63] emphasized the impact of pain on muscle recruitment patterns, particularly within the deep lumbar extensors, further contributing to compromised balance.

The observed decrement in functional balance in individuals with lumbar spondylosis holds clinical significance, particularly regarding the elevated risk of falls. Studies have consistently shown a correlation between lumbar spine disorders and an increased risk of falls, especially among older adults [64,65,66]. These findings underscore the urgency of addressing balance impairments as a crucial component of the comprehensive management of lumbar spondylosis. In summary, the substantial reduction in functional balance among individuals with lumbar spondylosis is not only anticipated but is also substantiated by a wealth of scientific literature. These results highlight the critical importance of addressing functional balance impairments in the holistic management of lumbar spondylosis, potentially enhancing patients’ ability to perform daily activities while mitigating the risk of falls.

The significant differences observed in key parameters such as ellipse area, A–P sway, and M–L sway under both eyes-open and eyes-closed conditions provide compelling reasons for justification and are in alignment with established research in the field [67,68]. These pathophysiological changes of lumbar spondylosis can significantly impact an individual’s ability to control their posture and maintain stability [69]. Consequently, the observed discrepancies in the limits of stability variables are consistent with the expected challenges in postural control faced by individuals with lumbar spondylosis [67,68]. Furthermore, muscle weakness and imbalances, particularly in the lumbar extensor muscles, are frequently observed in individuals with lumbar spondylosis [67,68]. Weakness in these crucial stabilizing muscles can impede the lumbar spine’s ability to withstand dynamic postural control tasks, further exacerbating the challenges of maintaining balance. This is in line with previous studies, such as Brumagne et al. [68], which demonstrated that individuals with chronic low back pain, a condition closely associated with lumbar spondylosis, often exhibit diminished lumbar extensor muscle endurance and strength, contributing to impaired postural control. Chronic pain, a hallmark symptom of lumbar spondylosis, introduces another layer of complexity to postural control [70]. Pain can lead individuals to adopt altered movement patterns, limit their range of motion, and reduce their ability to effectively shift their COP during postural adjustments [71]. Hlaing et al. [72] emphasized the influence of pain on postural control mechanisms, reinforcing the critical role of pain-related alterations in individuals with lumbar spondylosis [72]. In light of the substantial differences in limits of stability variables observed between individuals with lumbar spondylosis and healthy controls, there emerges a pressing clinical implication. These findings underscore the necessity for targeted interventions aimed at enhancing postural stability in individuals grappling with lumbar spondylosis. The potential benefits extend beyond mere functional improvement, as improved postural control may also mitigate the elevated risk of falls and related complications—a matter of paramount concern in this population.

In patients with lumbar spondylosis, the correlations among lumbar extensor endurance, functional balance, and limits of stability variables are limited. However, these findings still align with prior research in the field, underscoring their significance [38,41,73]. The positive correlation between lumbar extensor endurance and functional balance, and negative correlations between lumbar extensor endurance and limits of stability variables underscore the intricate interplay between lumbar extensor function, balance, and stability in individuals afflicted with lumbar spondylosis [41]. These findings align with a comprehensive understanding of the musculoskeletal system, particularly in the context of lumbar spine pathologies [41,74]. Lumbar extensor endurance, a key measure of the musculature supporting the lumbar spine, plays a vital role in stabilizing the spine during dynamic activities and maintaining an upright posture [75]. When individuals with lumbar spondylosis experience improvements in lumbar extensor endurance, it enhances their capacity to provide essential spinal stability during various functional tasks [75]. Functional balance, on the other hand, encompasses the ability to maintain equilibrium during daily activities, and it is highly reliant on the coordination and strength of the muscles involved [63]. The positive correlation between lumbar extensor endurance and functional balance suggests that as lumbar extensor endurance improves, individuals with lumbar spondylosis may experience enhanced postural control, contributing to a reduced risk of falls and improved overall functional performance [64]. Furthermore, the correlations observed in limits of stability variables, both with eyes open and eyes closed, indicate that improvements in lumbar extensor endurance can have a cascading effect on an individual’s ability to control their COP within their base of support. This is particularly vital for maintaining stability during dynamic movements and tasks.

Studies such as that by Unger et al. [76] and Standaert et al. [77] have emphasized the potential benefits of targeted interventions aimed at improving specific muscle groups, including lumbar extensors, in enhancing postural control and stability. Their findings align with our study’s results, highlighting the positive associations between lumbar extensor function and postural control in individuals with musculoskeletal conditions. In summary, the positive correlations observed between lumbar extensor endurance, functional balance, and limits of stability variables within the lumbar spondylosis group are consistent with the interconnected nature of musculoskeletal function in individuals grappling with lumbar spondylosis. These findings underscore the potential for targeted interventions focused on improving lumbar extensor endurance to have a positive cascading effect on functional balance and stability, ultimately contributing to improved quality of life for individuals with lumbar spondylosis.

### 4.1. Limitations of the Study

While this study offers valuable insights, several limitations must be acknowledged. First, the cross-sectional design limits the establishment of causality. Longitudinal studies could provide more insight into the temporal relationships among the studied variables. Second, the study’s generalizability may be constrained by the specific demographic characteristics of the participants and the single-center setting. Additionally, potential selection bias may exist, as participants with more severe lumbar spondylosis may be more likely to seek medical attention, potentially influencing the study’s findings. Furthermore, the reliance on self-report measures for pain intensity and functional disability introduces the possibility of response bias.

### 4.2. Strengths of the Study

Our study presents several strengths that bolster its significance in the context of lumbar spondylosis research. We employed well-established assessments, including the reliable Sorensen test for lumbar extensor muscle endurance, the Berg Balance Scale for functional balance evaluation, and a computerized stabilometric force platform for comprehensive limits of stability assessment. These assessments provided in-depth insights into the impact of lumbar spondylosis on various facets of spinal stability and overall low back health. Our meticulous diagnosis and characterization of the lumbar spondylosis group by experienced orthopedics, along with the inclusion of an age-matched healthy control group, ensured robust comparisons. Collectively, these strengths enhance the scientific rigor and relevance of our study, offering valuable insights into the functional limitations and rehabilitation requirements of individuals with lumbar spondylosis.

## 5. Conclusions

In this comprehensive study, we investigated lumbar extensor endurance, functional balance, and limits of stability in individuals with lumbar spondylosis, comparing them with age-matched healthy controls. Our findings revealed significant impairments in lumbar extensor endurance, functional balance, and postural stability among the lumbar spondylosis group. Moreover, positive correlations were observed between lumbar extensor endurance and functional balance, and negative correlations with stability, highlighting their interdependence within the lumbar spondylosis population. These results emphasize the importance of addressing lumbar extensor function, balance, and stability in the management of lumbar spondylosis, potentially enhancing daily functioning and reducing fall risk. However, the study acknowledges limitations and the need for further research to corroborate these findings and develop evidence-based interventions for this condition.

## Figures and Tables

**Table 1:** Physical and demographic characteristics of the participants.

Characteristics	Lumbar Spondylosis Group (n = 60)	Healthy Control Group (n = 60)	*p*-Value
Age (years) (mean ± SD)	59.73 ± 10.08	58.98 ± 8.67	0.826
Sex, (male: female)	32:28	33:27	0.975
BMI (kg/m^2^) (mean ± SD)	24.64 ± 3.24	23.83 ± 2.32	0.416
Duration of symptoms (months)	12.33 ± 4.56	-	-
Pain intensity (VAS scale) 0–100 mm	57.66 ± 22.11	-	-
Functional disability (ODI score) 0–50	22.63 ± 8.09	-	-

SD, standard deviation; BMI, body mass index; VAS, visual analogue scale; ODI, Oswestry Disability Index.

**Table 2:** Comparisons of lumbar extensor endurance, functional balance, and limits of stability variables between the groups.

Characteristics	Lumbar Spondylosis Group (n = 60) (mean ± SD)	Healthy Control Group (n = 60) (mean ± SD)	*p*-Value
Lumbar extensor endurance (seconds)	23.06 ± 8.38	52.45 ± 11.48	<0.001
Functional balance (BBS scores, 0 to 56)	48.36 ± 3.26	53.34 ± 2.48	<0.001
Limits of stability variables—Eyes open			<0.001
Ellipse Area (mm^2^) A–P sway (mm) M–L sway (mm)	337.07 ± 128.12	176.94 ± 63.87	
	6.43 ± 2.16	4.23 ± 1.01	
	3.38 ± 0.89	2.12 ± 0.32	
Limits of stability variables—Eyes closed			<0.001
Ellipse Area (mm^2^) A–P sway (mm) M–L sway (mm)	512.38 ± 110.26	256.12 ± 98.87	
	8.32 ± 2.92	5.12 ± 2.08	
	5.23 ± 1.96	3.99 ± 1.98	

SD, standard deviation; BBS, Berg Balance score; A–P sway, anterior to posterior sway; M–L sway, medial to lateral sway.

**Table 3:** Correlation Analysis of Lumbar Extensor Endurance with Functional Balance and Limits of Stability Variables in Lumbar Spondylosis Patients.

Characteristics	Lumbar Extensor Endurance (seconds) (r)
Functional balance (BBS scores, 0 to 56)	0.46 **
Limits of stability variables—Eyes open	
Ellipse Area (mm^2^) A–P sway (mm) M–L sway (mm)	−0.38 **
	−0.41 **
	−0.43 **
Limits of stability variables—Eyes closed	
Ellipse Area (mm^2^) A–P sway (mm) M–L sway (mm)	−0.49 **
	−0.47 **
	−0.45 **

SD, standard deviation; BBS, Berg Balance score; A–P sway, anterior to posterior sway; M–L sway, medial to lateral sway; **, level of statistical significance ≤ 0.01.

## Data Availability

The dataset employed in this study is accessible through the corresponding author, RSR, and will be made available upon formal request.
